# Bardet–Biedl syndrome: beyond the cilium

**DOI:** 10.1007/s00467-007-0435-0

**Published:** 2007-07-01

**Authors:** Jonathan L. Tobin, Philip L. Beales

**Affiliations:** Molecular Medicine Unit, UCL Institute of Child Health, 30 Guilford Street, London, WC1N 1EH UK

**Keywords:** Bardet–Biedl, Cystic kidneys, Primary cilia, Wnt signalling, Sonic hedgehog

## Abstract

The Bardet–Biedl syndrome (BBS) is a significant genetic cause of chronic and end-stage renal failure in children. Despite being a relatively rare recessive condition, BBS has come to prominence during the past few years owing to revelations of primary cilia dysfunction underlying pathogenesis. The study of this multi-system disorder, which includes obesity, cognitive impairment, genito-urinary tract malformations and limb deformities, is beginning to reveal insights into several aspects of mammalian development and organogenesis. Involvement of BBS proteins in disparate pathways such as the non-canonical Wnt and Sonic Hedgehog pathways is highlighting their interplay in disease pathogenesis. Here we review the recent developments in this emerging field, with the emphasis on the renal component of the syndrome and potential future directions.

## Overview of Bardet–Biedl syndrome

The unlikely ensemble of signs that the Bardet–Biedl syndrome presents has bewildered doctors for over a hundred years. In 1865 Laurence and Moon reported the first case of an obese, visually impaired girl with intellectual disabilities [1]. This triad of features was extended in the 1920s by two independent reports by George Bardet and Artur Biedl describing the additional characteristics of polydactyly and hypogenitalism [2, 3]. These remain the cardinal features of Bardet–Biedl syndrome (BBS), but further manifestations of the disease have since been recognised. Polycystic kidneys are the most likely cause of premature death from the syndrome, combined with complications caused by overweight, including type II diabetes, hypertension and hypercholesterolaemia [4] (see Table 1 and Fig. 2 for diagnostic criteria). This review summarises some key recent findings in BBS research, focusing on the renal components of the syndrome. In the light of recent revelations we speculate on the aetiology of several aspects of the syndrome that may lie beyond the cilium.

**Table 1:** Diagnostic criteria for BBS

	Percent prevalence (modified from [4])	Comment
**Primary features**		
Rod-cone dystrophy	93%	Other ocular defects included: astigmatism, strabismus, cataracts, colour blindness, macular oedema and degeneration, and optic atrophy
Post-axial polydactyly	69%	Present on all four limbs in 21% of patients, only hands in 9% and only feet in 21%. Brachydactyly present in 46% and syndactyly in 9% of patients
Truncal obesity	72%	Mean BMI in males was 31.5 kg/m^2^, in females it was 36.6 kg/m^2^
Hypogonadism	98%	Males had hypogenitalism and 8% had maldescended testes. Most women reported irregular menstrual cycles
Renal anomalies	24% (only 52% of patients had undergone renal examination)	Renal parenchymal cysts (10%), calyceal clubbing (10%), foetal lobulation (12%), scarring (12%), dysplastic kidneys (5%), unilateral agenesis (4%), renal calculi (2%), vesicoureteric reflux (9%), bladder obstruction (4%), hydronephrosis (4%), horseshoe kidney (2%), ectopic kidney (2%)
**Secondary features**		
Speech disorder/delay	54%	
Developmental delay	50%	52% showed delay in walking of up to 1 year, speech delayed by up to 2 years in 47%, delay in pubescence in 31% (all males)
Behaviour	33%	Emotional immaturity, outbursts, disinhibition, depression and lack of social dominance, obsessive compulsive behaviour
Ataxia/imbalance	40%	Abnormal gait reported in 33% of patients (see [5])
Diabetes mellitus	6%	
Congenital heart defects	7%	Included: aortic stenosis, patent ductus arteriosis, cardiomyopathy
Liver disease		Hepatic fibrosis
Hearing loss	21%	Predominantly conductive but some sensorineural
Facial features		Deep-set eyes, hypertelorism, long philtrum, thin upper lip, anteverted nares, prominent forehead with male early-onset balding
Situs inversus	Unknown	See [6]
Hirschprung disease	Unknown	See [6]
Polyuria/polydipsia		May be present in the absence of renal abnormality
Dental crowding		Also includes: high arched palate, hypodontia, small roots
Anosmia	~60%	See [11]

**Fig. 1:**
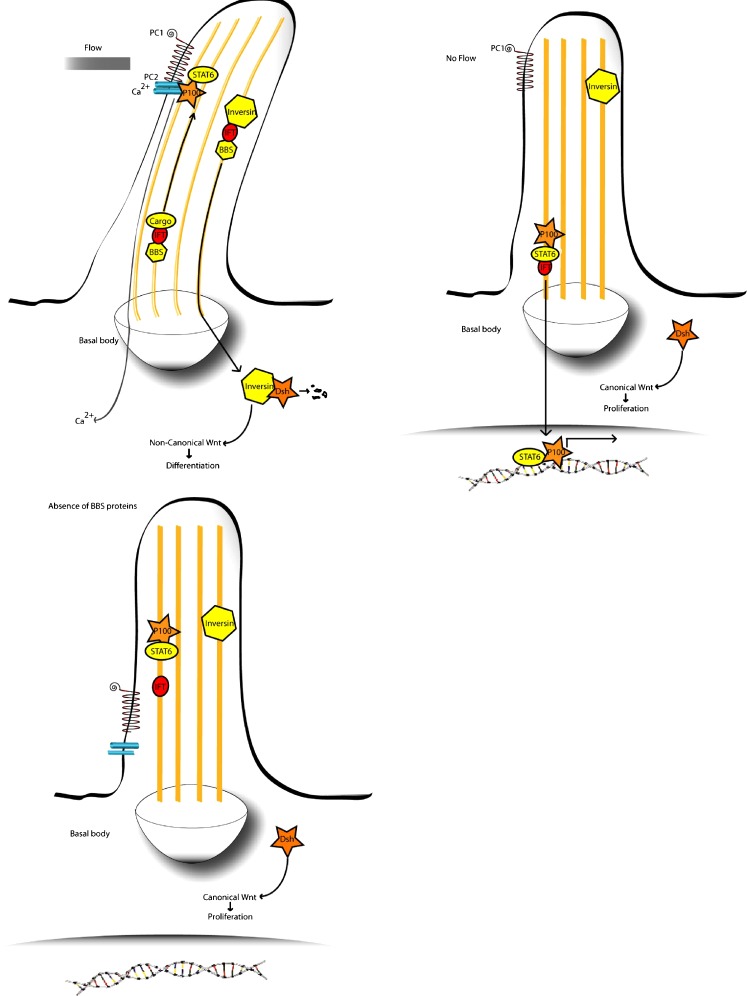
Putative pathomechanism for renal cystic hyperplasia in BBS. *Top left* Urine flow through the kidney tubule causes an influx of Ca^2+^ ions through polycystin 2 (*PC2*), while polycystin 1 (*PC1*) anchors the transcriptional complex of P100 and STAT6 in the cilium. Concurrently, inversin is translocated to the nucleus, where it targets cytoplasmic Dishevelled for destruction, activating the non-canonical pathway and causing cells to differentiate. The ciliary localisation of PC1/2 and inversin may be dependent on BBS proteins. *Top right* Absence of urine flow reduces Ca^2+^ influx and causes release of P100 and STAT6, allowing them to enter the nucleus to activate transcription. It also prevents translocation of inversin to the cytoplasm, maintaining the cytoplasmic pool of Dishevelled, which activates the canonical Wnt pathway and causes proliferation. *Bottom left* A lack of BBS protein function may inhibit proper transport of PC1/2 to the distal tip of the cilium and also prevent translocation of inversin to the cytoplasm. This would cause inappropriate activation of STAT6 target and maintenance of cytoplasmic Dishevelled, leading to unregulated cell proliferation. Additionally, lack of BBS protein function could disturb planar cell polarity (PCP). The combination of disorganised cell polarity and cell division could cause the various abnormalities seen in BBS

Although BBS is considered to be a developmental disorder, the only clues in utero may be hexadactyly and hyperechoic kidneys [7]. Birth weight tends to be normal, but rapid weight gain begins after the first year, probably due to hyperphagia rather than metabolic abnormalities [8]. Language acquisition may be delayed until the child is 4 years of age. Diagnosis of BBS is often only established once the vision begins to degrade. Night blindness manifests when the child is around 8 years and is followed by loss of peripheral vision, usually progressing to significant blindness by 15 years [4].

## Twelve BBS genes identified

Early linkage studies revealed a high level of heterogeneity, culminating in the discovery of not one but, so far, 12 genes (summarised in Table 2). When mutated, any of these genes can cause the plethora of BBS features. As there appears to be no correlation between genotype and phenotype, it has been hypothesised that the different BBS proteins are functioning in a common cellular process [9]. In 2003 Ansley et al. proposed that BBS was caused by a dysfunction of the cilium and its associated basal body, an organelle derived from the centriole acting as a nucleation site for ciliary axonemal microtubules [10]. They discovered *BBS8* and showed that its protein localises to the centrosome and basal body. BBS8 interacts with peri-centriolar material 1 (PCM1), a protein important for ciliogenesis. Further, in the nematode *C. elegans*, *bbs8* is regulated by the transcription factor *daf-19* (an RFX-type transcription factor), a regulator of genes involved in ciliogenesis and transport [10].

**Table 2:** BBS genes identified so far (*IFT* intraflagellar transport)

Gene	Method of discovery	Chromosomal location	Cellular localisaiton	Domains	Putative function	Reference
*BBS1*	Linkage analysis	11q13	Basal body/cilium	None	Cilia function	[11]
*BBS2*	Positional cloning	16q21	Basal body/cilium	None	Cilia function/flagellum formation	[12]
*BBS3/ARL6*	Linkage analysis	3p12-q13	Basal body/cilium	GTP-binding	Vesicle trafficking	[13]
*BBS4*	Positional cloning	15q23	Pericentriolar/basal body	TPR/PilF	Microtubule transport	[14]
*BBS5*	Comparative genomics	2q31	Basal body/cilium	DM16 DUF1448	Cilia function/flagellum formation	[15]
*BBS6/MKKS*	Mutation analysis	20p12	Basal body/cilium	TCP1 chaperonin	Cilia function/flagellum formation	[16]
*BBS7*	Similarity to BBS2	4q32	Basal body/cilium	TPR/PilF	IFT particle assembly	[17]
*BBS8/TTC8*	Similarity to BBS4	14q31	Basal body/cilium	TPR/PilF	IFT particle assembly	[17]
*BBS9/B1*	Homozygosity mapping with SNP arrays	7p14.3	Unknown	COG1361 membrane biogenesis	Unknown—expressed in bone cells	[18]
*BBS10*	SNP arrays	12q21.2	Unknown	TCP1 chaperonin	Unknown	[19]
*BBS11/*						
* TRIM32*	SNP arrays	9q31-34.1	Unknown	RING WD40 NHL Barmotin B-Box	E3 ubiquitin ligase	[20]
* BBS12*	SNP arrays	4q27	Unknown		Type II chaperonin	[21]

Since this initial association of BBS8 with the basal body, putative roles for other BBS proteins are being established. These are summarised in Table 2. BBS1, 2, 4, 5, 6, and 8 all localise to the basal body and peri-centriolar region. In *C. elegans* BBS7 and BBS8 localise to the base of the cilium and are transported along the axoneme of sensory neurons [17]. BBS4 functions in the microtubule apparatus and interacts with subunits of dynactin, implying a role in retrograde intraflagellar transport (IFT) [14]. BBS6, 10, and 12 are likely chaperonins, which may facilitate protein folding and together account for one third of all cases [21]. Why mutations in chaperonins should cause the features of BBS is, as yet, unknown, but it could be due to aberrant folding of proteins essential for IFT or ciliogenesis.

## Aetiology lies in the cilium

Cilia are ancient eukaryotic organelles that project from the cell surface. They fall into two classes: motile and immotile. Motile cilia consist of an outer ring of nine doublets of microtubules surrounding an inner pair—the “9 + 2” arrangement. Radial spokes link the outer ring doublets to the inner doublet, and dynein motor proteins, spanning adjacent outer doublets, power the beating of the cilium. This beating motion is used to drive fluid flow in the respiratory tract and oviduct, and propels spermatozoa.

The features of BBS do not, in the main, appear to reflect a defect in motile cilia (except for aflagellate spermatozoa). Rather, it seems that the immotile sensory (also called primary) cilia are predominantly affected. These consist of a “9 + 0” arrangement of microtubule doublets and lack the central microtubule pair present in motile cilia. Until recently, primary cilia were considered to be evolutionary vestiges, with no obvious function. Now their role in vertebrate physiology is finally being elucidated; many key signalling pathways rely on the cilium for their function. The list of molecular pathways with key components localising to the primary cilium includes: PDGFRα growth factor signalling [22], hedgehog signalling [23], epidermal growth factor signalling [24], and 5-HT_6_ serotonin signalling [25]. See Marshall and Nonaka (2006) for a more comprehensive list of cilia-dependent signalling pathways [26]. The study of BBS is improving our understanding of the importance and complexity of these organelles in development and homoeostasis.

It is now thought that BBS proteins participate in IFT [17, 27]. Because cilia lack ribosomes, all proteins involved in their construction and function must first be imported and then shuttled along the ciliary axoneme by a raft of proteins [9]. Cargo bound for the distal end of the cilium is loaded in the cytoplasm at the transition zone at the base of the cilium. Anterograde transport is facilitated by the microtubule motor protein kinesin-2. Retrograde transport back to the cytoplasm deploys the dynein–dynactin motor complex. See Blacque and Leroux for a detailed review of the roles of BBS proteins in IFT [28].

Some BBS proteins are thought to function as adaptors, helping to load cargo proteins at the proximal cytoplasmic end for carriage to the distal tip [17]. BBS proteins are also thought to be required for retrograde transport, where proteins are shuttled back to the cytoplasm for recycling. Figure 1 shows how the IFT processes are coordinated in the cilium. Mutations in a BBS gene reduce or abolish their protein function, disrupting IFT. This has been demonstrated in *C. elegans*, where abrogation of *bbs7* or *bbs8* both reduces the length of cilia in neurons (structure) and disrupts IFT machinery, leading to improper transport of kinesin and other IFT components along the cilium (function) [17].

**Fig. 2:**
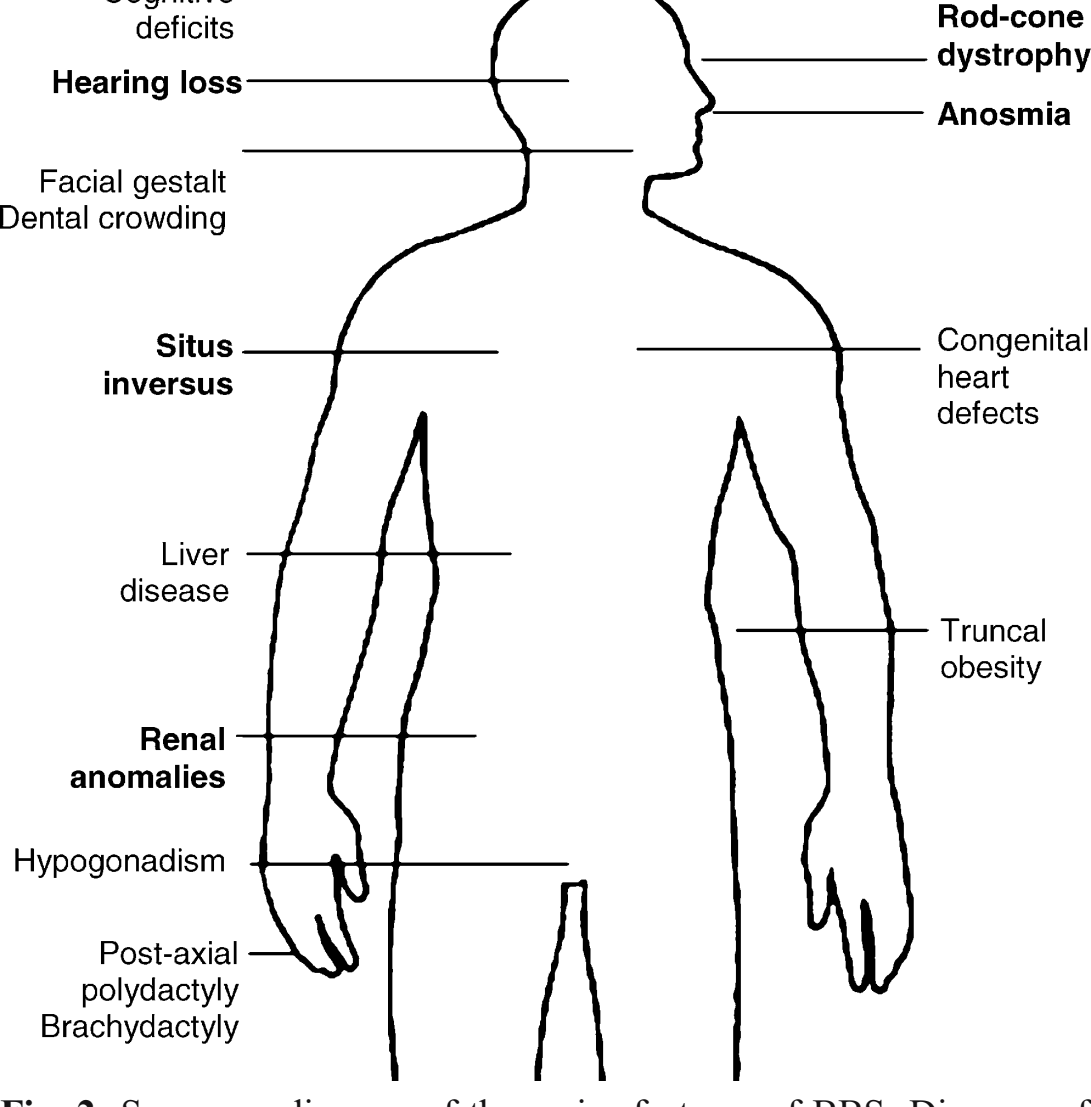
Summary diagram of the major features of BBS. Diagram of major features of BBS. Features directly attributable to defects in ciliary function are marked in *bold*

Because BBS proteins are not absolutely required for ciliary function, mutations in BBS genes do not cause as dramatic an effect as mutations in core IFT proteins such as *Kif3a*, which die at 10 days after fertilisation [29]. As such, the clinical phenotypes of BBS mutants are less severe than one might expect if IFT were completely abrogated. Some BBS features can be readily attributed to deficient IFT: retinitis pigmentosa (RP) can result from transport defects in the connecting cilium of the rod and cone cells in the retina, leading to progressive degeneration of the photoreceptors [30]. Figure 2 summarises these features. Anosmia is common amongst patients, owing to flaws in olfactory cilia [11]. Infertility has been reported in *Bbs* null mice, where immotility was attributed to failure of the spermatozoa flagella to develop [31]. Some BBS patients have situs inversus, a complete reversal of visceral laterality, arising from defective cilia in the early embryonic node. Perhaps the best characterised feature of a presumed ‘ciliopathy’ is the cystic kidneys, with their aetiology in aberrant ciliary function.

## Renal disease is a major cause of mortality in BBS

**Fig. 3:**
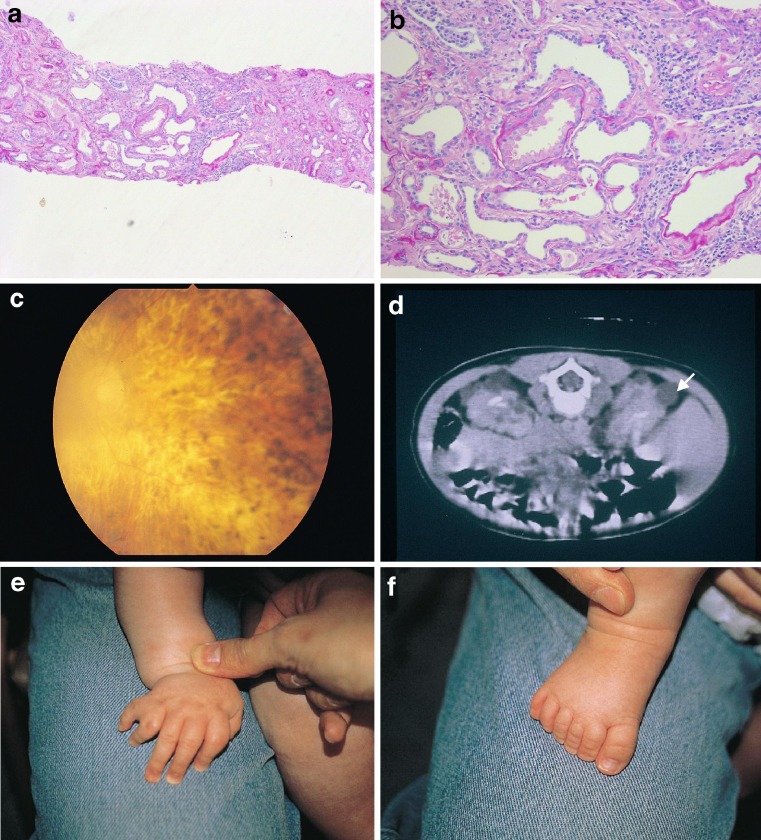
Examples of renal histopathology in BBS patients. **a**, **b** Low and high power micrographs showing tubular dilatation in a biopsy from a BBS patient’s kidney (Histopathological sections courtesy of Dr. Neil Sebire, Great Ormond Street Hospital). **c** Fundoscopy showing retinitis pigmentosa with cataract. **d** Abdominal CT scan documenting cystic kidneys (*arrowed*). **e**, **f** Post-axial polydactyly in a hand and foot from the same child with BBS

Beales et al. (1999) reported imaging 59 BBS patients and found that 46% had abnormal renal structure but only 5% had functional impairment at the time of scanning [4]. Large, hyperechoic kidneys can be detected prenatally by ultrasound but are not necessarily a predictor of subsequent dysfunction [7]. Examples of renal histopathology observed in BBS patients are shown in Fig. 3.

Two reports in the 1970s described seven patients with cystic spaces communicating with collecting ducts [32, 33]. O’Dea (1996) conducted a prospective cohort study on 38 patients in Newfoundland, where the disease is ten-times more prevalent than in mainland Europe or America due to founder effects [34]. They found that 96% of patients had foetal lobulation, calyceal cysts, diverticula, or clubbing. Twenty-five percent of patients suffered functional impairment, fatally in two patients with end-stage renal disease. Another patient died from metastatic renal cancer.

Beales and colleagues documented an excess of relatively early-onset renal cell carcinomas in obligate carriers of BBS mutations. Furthermore, they detected loss of heterozygosity in tumour sections at several BBS loci [35]. Ersoy et al. (2005) reported a 20-year-old patient who had undergone renal transplantation who then developed primary central nervous system lymphoma. They suggested that BBS patients should be carefully monitored for developing malignancies [36].

In a cohort of 20 patients in Newfoundland, Harnett and colleagues identified three with end-stage renal failure [37]. Fourteen (70%) patients could not concentrate urine above 750 mosmol/kg body weight, even after vasopressin treatment. Upon ammonium chloride administration, urinary pH fell in only 13 patients (65%). Structural abnormalities tend to be common, with varying degrees of functional impairment. Alton and McDonald asserted that 30% of BBS patients die from uraemia [32]. Chronic renal dialysis, or transplantation, has been the only successful mode of managing these problems for most patients. Langer and colleagues (2005) presented details of a 57-year-old patient who had undergone cadaveric kidney transplantation. Despite developing subsequent pneumonia, cytomegalovirus and scabies infections post-operatively, he was fully rehabilitated within 18 months [38].

Currently, in our own cohort, approximately 10% of BBS patients have undergone transplantation (unpublished data). An analysis of the long-term outcome of renal transplantation in BBS is underway, but initial observations suggest that children manage very well in the immediate-to-medium post-operative period (unpublished data). Modern steroid-sparing, anti-rejection regimens should be implemented to optimise glycaemic control and avoid exacerbation of obesity.

## Ciliary origins of kidney disease in BBS

Renal abnormality in BBS is thought to arise as a direct result of compromised cilia function, although this has yet to be proven. The origin of this hypothesis lies in the oak ridge polycystic kidney disease (*or*^*pkd*^) mouse mutant, which has dilated proximal tubules and cysts, and its cilia are short and poorly formed [39]. The mutation was mapped to the gene encoding polaris, a protein required for assembly of renal cilia [40]. Mutations in the *PKHD1* gene cause autosomal recessive polycystic kidney disease (ARPKD). The protein product, polyductin, co-localises with polycystin 2 at the base of the cilium [41].

In the nephron, cilia projecting into the lumen from the apical surface of epithelial cells bend in response to urine flow. The degree of bending is directly related to the fluid pressure and causes the opening of calcium channels in the cilium. The influx of calcium ions is proportional to the flow and activates intracellular signalling pathways thought to regulate the cell’s decision either to proliferate or differentiate. These calcium channels are formed by a complex of polycystin 1 and polycystin 2 (PC1 and PC2), encoded by the *PKD1* and *PKD2* genes, respectively, which cause autosomal dominant polycystic kidney disease (ADPKD) when mutated [42]. PC1 binds to the transcription factor STAT6 and its co-factor p100. Upon mechanical shearing caused by urine flow, PC1 disassociates from STAT6, which travels down the cilium to enter the nucleus where it activates target genes [43]. It remains to be discovered whether BBS proteins are involved in the transport and/or localisation of these ciliary proteins.

## Wnt signalling during kidney development may be regulated at the cilium

Recent evidence is emerging of additional regulatory roles for the cilium. It seems that the cilium may provide a regulatory switch between the canonical and non-canonical Wnt pathways [44]. In the Wnt pathway, diffusible extra-cellular Wnt ligands bind to their respective membrane-bound Frizzled receptors, leading to the activation of intracellular Dishevelled. If this then activates β-catenin, the canonical pathway is effected and is associated with re-entry into the cell cycle and proliferation. This pathway is required for branching morphogenesis during embryonic kidney development. Canonical Wnt signalling is dysregulated in *Invs* mice kidneys, which have a mutation in the gene encoding the ciliary protein inversin. In humans this gene (*NPHP2*) causes nephronophthisis (the most common autosomal recessive cause of early-onset renal failure) type II when mutated, which comprises interstitial cell infiltration, with fibrosis, and duct proliferation with cyst development [45]. Interestingly, the kidneys are massively enlarged in *NPHP2* mutated mice and patients. Inversin is activated by tubular fluid flow and targets Dishevelled for destruction, inhibiting the canonical Wnt pathway and driving cells down the non-canonical Wnt or PCP pathway towards differentiation and polarisation throughout the plane of the tubular epithelium.

In support of this hypothesis, we demonstrated that BBS proteins are involved in the PCP pathway. Of *Bbs4* null mice, 14% display anterior neural tube defects indicative of PCP dysfunction [30]. In adulthood, null mice have disorientated stereociliary bundles on the outer hair cells of the cochlea, and mice heterozygous for both *Looptail* (also known as *Vangl2*, a “core” PCP gene) and *bbs4* have phenotypes reminiscent of PCP mutants, whilst both single heterozygotes are normal. Confirmation of the interaction was made in zebrafish by the injection of a *bbs4* morpholino on a PCP mutant background and observing enhancement of the PCP phenotype.

A subsequent study by Fischer and colleagues suggested that this interaction may have implications for BBS-related cystogenesis. They measured the mitotic orientation of cells taken from the kidney tubules of a rat model of polycystic kidney disease and found them to be significantly disorientated [46]. They attributed this to mis-expression of polyductin (also called fibrocystin), a ciliary membrane protein mutated in ARPKD. As mitotic spindle orientation is regulated by the PCP pathway, aberrations caused by BBS protein dysfunction could cause similar mitotic defects in BBS kidneys. These results point to a need for hyperplasia caused by overactivity of the canonical Wnt pathway, combined with concomitant defects in planar cell polarisation.

## BBS proteins in renal pathogenesis

So far, the precise role of BBS proteins in renal pathogenesis is unclear. It is possible that BBS proteins are involved in the transport of proteins such as the polycystin ion channels and polaris to the distal end of the cilium, where they function as components of the sensory apparatus.

It is also possible that BBS proteins are somehow regulating the function of inversin, which, in turn, is involved in the selection of canonical versus non-canonical Wnt pathways. If BBS proteins are absent, inversin function may be compromised, with the balance shifted in favour of the canonical pathway and promoting cystic hyperplasia. Disrupted PCP activity could be associated with a disordered renal epithelium. This, combined with a concomitant boost to canonical Wnt signalling, might explain why some BBS patients show complex renal involvement; such as mesangial cell proliferation, cystic dilation of tubules, and corticomedullary cysts [47].

## The ciliary plexus

It is emerging that the cilium is indispensable for certain key developmental signalling cascades. It is now becoming clear that many discrete pathways converge at, or are regulated by, the cilium. Park et al. recently showed that defects in the PCP proteins Inturned and Fuzzy cause defects in ciliogenesis, with concomitant effects on Sonic Hedgehog (Shh) signalling [48].

Another ciliopathy, oro-facial digital type I (OFD1), shares some cardinal features with BBS, notably cystic kidneys, polydactyly and craniofacial dysmorphology. Ferrante et al. (2006) showed that when mutated this protein causes misregulation of downstream Shh pathway targets in the neural tube [49]. In the developing limb the expression patterns of Gli3 and Patched1, direct Shh targets are unaltered; whilst various Hox genes essential for digital patterning are disrupted. This does not indicate that Gli3 function is unimpaired—Shh patterning depends on the balance between Gli3’s active and repressive forms, not the overall level of Gli3 transcript. Several other studies have shown that defects in ciliary transport cause an imbalance between active and repressive forms of Gli3, particularly when exposed to Shh protein [50–52].

BBS patients have several hallmarks of defective Shh signalling, polydactyly being the most obvious amongst them. Additionally, many patients have agenesis or hypoplasia of the corpus callosum, and a single central incisor, both elements of the holoprosencephaly spectrum (personal observations). It is perplexing why ciliopathies such as BBS, Meckel syndrome, and OFD show features of sonic abnormalities, whilst others, such as polycystic kidney disease (PKD) and Senior–Loken syndrome (nephronophthisis with retinal degeneration) do not.

Support for the involvement of the cilium in regulating both Shh and PCP pathways in development was recently demonstrated by Park and colleagues [48]. It was also recently shown that the Gli transcription factors which act downstream of Shh localise to the cilium too, and require IFT for their processing and transcriptional activity [23]. Interruption of the activity of Gli3 represses the activation of target genes responsible for renal morphogenesis (such as *Pax2* and *Sall1*) and results in decreased branching morphogenesis and dilated tubules [53]. Together, these observations may help account for some of the renal tissue abnormalities in BBS.

If BBS proteins are essential for all the above-mentioned signalling pathways and ciliary processes, one would expect the clinical phenotype to represent full loss of all involved molecules. However, BBS proteins implicated in IFT seem to be partially dispensable. In *C. elegans*, knock-out of BBS7 and BBS8 destabilises transport of IFT complexes but does not abrogate function altogether [54]. Using microtubule gliding assays, Pan et al. recently proposed that BBS7 and BBS8 act as a molecular bridge, holding the IFT particle complexes A and B together [55]. This holding force seems to be necessary to coordinate transport along the distal-most portion of the cilium. A lack of BBS protein function could result in disorganised shuttling of proteins whose distal localisation in the cilium is critical for proper function. Proteins transported in this way may include the polycystins and sonic hedgehog pathway components such as smoothened and Gli.

## Not all cardinal features can be attributed to the cilium

Ciliary dysfunction currently fails to explain, directly, hypogenitalism, polydactyly, mental retardation, or obesity in BBS. Nonetheless, we can postulate several mechanisms. For example, development of the genitalia and gonads is partly controlled by Shh and bone morphogenetic protein 4 (BMP4) [56]. It is possible that misregulation of the Shh pathway is partly responsible, in combination with endocrine malfunction.

Post-axial polydactyly is present in 70% of patients. It is known that cells of the developing limb bud, in both ectoderm and mesenchyme, are ciliated [23]. Along with the known reliance of the Shh pathway on cilia, it is tempting to speculate that aberrant Shh signalling in the limb bud is the cause of the limb deformities seen in BBS. Post-axial polydactyly is also a primary feature of Pallister–Hall syndrome, a condition caused by a mutation in Gli3 [57].

The aetiology of obesity in BBS is more problematic although we do know that patients’ resting metabolic rate is normal [8]. Anecdotal reports hint that appetites are difficult to satiate, implicating a defect in the satiety centre of the hypothalamus. Like IFT, fast-axonal transport (FAT) is microtubular based and relies on dynein motors [58]. BBS proteins could play a part in transducing information about the satiety level to the neuronal cell bodies. However, there are no published data supporting a role for BBS proteins in FAT—as such, this is highly speculative. In *C. elegans* it has been shown that BBS proteins function in ciliated neurons that sense external and internal nutrient levels. *bbs1* mutants act synergistically to enhance accumulation of lipids in a *C. elegans* mutant with impaired β-oxidation of fatty acids [59].

Despite progress in the identification of pathways affected in BBS, it is proving difficult to recognise the point of interaction and, in particular, the components at fault. Nonetheless, it will be important to determine these factors, as they may reveal therapeutic targets. For example, two other centrosomally localised proteins—tuberose sclerosis 1 and tuberose sclerosis 2 (TSC1 and TSC2)—cause renal cysts when their genes are mutated. As both proteins inhibit mTOR, a kinase controlling cell proliferation, it is possible to reduce cyst growth by treatment with the mTOR inhibitor rapamycin. Furthermore, it has been shown that the mTOR pathway is regulated by polycystin 1 and that ADPKD patients’ cysts can be ameliorated by rapamycin treatment [60]. Determining if BBS proteins impinge on these pathways in the kidney may provide a remedy that is already well characterised.

## Beyond the cilium?

Unexpected new BBS genes (*BBS9-12*) are being discovered that do not show any intuitive relationship with the cilium or IFT. It is, therefore, likely that BBS proteins have extra-ciliary roles. Recently identified BBS genes include *BBS9* (also called *B1*), a parathyroid hormone-responsive protein [18]. *BBS10* and *BBS12* show similarity to *BBS6*, and are likely to encode chaperonins with the potential to fold nascent proteins. *BBS10* is a major pathogenic locus, accounting for 20% of cases [19]. It has been suggested that *BBS6*, *BBS10*, and *BBS12* may act redundantly [21].

*BBS11* (also called *TRIM32*) mutations have been identified only in a single consanguineous Bedouin family [20]. It encodes an E3 ubiquitin ligase and may be involved in protein degradation. Interestingly, TRIM32 has recently been shown to interact with a protein important for the sumoylation pathway; Piasy [61]. Whilst ubiquitination degrades proteins, sumoylation stabilises them. BBS11 may be involved in the regulation of the stability and lifetime of proteins associated with the basal body and IFT. Subcellular localisation for these newly discovered proteins is unconfirmed at present, and their function in wild type organisms has yet to be elucidated.

So far, we know that the PCP, the Shh, and, possibly, the canonical Wnt (via inversin) pathways rely, at least in part, on the cilium for their function and are intersected by the BBS proteins. The future direction of research in this field will involve the precise definition of the molecules that are being regulated by BBS protein malfunction, how this impacts on their downstream effectors, and whether this knowledge could lead to any therapeutic intervention.

We must determine why BBS proteins with different functions produce a syndrome with such a consistent phenotype. Conversely, why is it that individuals with the same mutations display such variable phenotypes? Steps towards answering this question were made by the recent discovery of a novel gene, *MGC1203*, which contributes epistatically to BBS mutations and can enhance the phenotype, at least in zebrafish embryos [62]. The combined input of epistatic and mechanistic studies, derived from multiple animal models, should bring us closer to understanding this complex disease.

## Conclusion

BBS is a rare pleiotropic syndrome, the study of which has been both challenging and informative well beyond the scope of the disease itself. So far, the cilium has been held responsible for the bulk of the pathology, but the precise mechanisms and pathways involved are only just being revealed. The PCP and, potentially, sonic hedgehog pathways may contribute, in some part, to our understanding of these problems. The biology of polycystic kidney disease is in accord with our current understanding of BBS and may explain the associated renal dysgenesis. In turn, this is likely to involve imbalanced Wnt signalling, but further direct functional evidence is required.
